# A T cell-intrinsic function for NF-κB RelB in experimental autoimmune encephalomyelitis

**DOI:** 10.1038/s41598-021-99134-x

**Published:** 2021-10-04

**Authors:** Guilhem Lalle, Raphaëlle Lautraite, Allison Voisin, Julie Twardowski, Pierre Stéphan, Marlène Perrin-Niquet, Ramdane Igalouzene, Saidi M. Soudja, Julien C. Marie, Marc Vocanson, Nilushi De Silva, Ulf Klein, Sankar Ghosh, Yenkel Grinberg-Bleyer

**Affiliations:** 1 Cancer Research Center of Lyon, UMR INSERM 1052, CNRS 5286, Université Claude Bernard Lyon 1, Labex DEVweCAN, Centre Léon Bérard, Lyon, France; 2 CIRI-Centre International de Recherche en Infectiologie, INSERM, U1111, Université Claude Bernard Lyon 1, Ecole Normale Supérieure de Lyon, CNRS UMR 5308, Lyon, France; 3 Immunity and Cancer Department, Institut Curie, Paris-Sciences-Et-Lettres Research University, INSERM U932, Paris, France; 4 Division of Haematology and Immunology, Leeds Institute of Medical Research at St. James’s, University of Leeds, Leeds, UK; 5 Department of Microbiology and Immunology, College of Physicians and Surgeons, Columbia University, New York, NY 10032 USA

**Keywords:** Cell biology, Immunology

## Abstract

NF-kappaB (NF-κB) is a family of transcription factors with pleiotropic functions in immune responses. The alternative NF-κB pathway that leads to the activation of RelB and NF-κB2, was previously associated with the activation and function of T cells, though the exact contribution of these NF-κB subunits remains unclear. Here, using mice carrying conditional ablation of RelB in T cells, we evaluated its role in the development of conventional CD4^+^ T (Tconv) cells and their function in autoimmune diseases. RelB was largely dispensable for Tconv cell homeostasis, activation and proliferation, and for their polarization toward different flavors of Thelper cells in vitro. Moreover, ablation of RelB had no impact on the capacity of Tconv cells to induce autoimmune colitis. Conversely, clinical severity of experimental autoimmune encephalomyelitis (EAE), a mouse model of multiple sclerosis (MS) was significantly reduced in mice with RelB-deficient T cells. This was associated with impaired expression of granulocyte–macrophage colony-stimulating factor (GM-CSF) specifically in the central nervous system. Our data reveal a discrete role for RelB in the pathogenic function of Tconv cells during EAE, and highlight this transcription factor as a putative therapeutic target in MS.

## Introduction

Classically split into two separate activation pathways, the NF-κB family is composed of 5 subunits that all share a Rel homology domain and associate as homo- or heterodimers to modulate gene expression^1^. In T cells, stimulation of the canonical pathway is classically triggered by the engagement of T-cell receptor (TCR)/CD28 molecules, which drives the activation of a Carma1-Bcl-10-Malt-1/inhibitor of κB kinase (IKK)α/β/γ cascade. This, in turn, leads to the degradation of IκBα and the nuclear translocation of NF-κB1 (p105/p50, encoded by *Nfkb1*), RelA (or p65, encoded by *Rela*) and c-Rel (encoded by *Rel*) proteins^2^. The alternative NF-κB pathway is generally triggered by the engagement of different members of the tumor necrosis factor receptor (TNFRSF) superfamily, and consists in the activation of NIK, which phosphorylates IKKα, driving the processing of NF-κB2 (p100/p52, encoded by *Nfkb2*) and culminating in the nuclear translocation of p52 and RelB (*Relb*) subunits. Importantly, it is now clearly established that canonical and alternative subunits can dimerize with each other under certain conditions, conferring novel functions to NF-κB and adding another layer of complexity to its signaling^3–5^. In addition, RelB was suggested to repress gene transcription at given loci by recruiting chromatin modifiers, independently of other NF-κB subunits; thereby promoting immune tolerance under certain circumstances^6–8^. A number of studies have reported the multiple functions of NF-κB in inflammation and immunity. In particular, it was suggested to exert critical roles in the development and homeostasis of CD4^+^ T lymphocytes^9,10^.

CD4^+^ T cells are pivotal actors of adaptive immune responses. Following stimulation, naïve conventional CD4^+^ T (Tconv) cells can polarize into different flavors of Thelper (TH) cells depending on the cytokine milieu to mount protective responses against specific classes of pathogens^11^. In addition to these protective roles, TH cells are involved in the pathogenesis of different immune disorders, such as allergy or autoimmunity. For instance, TH1 and TH17 cells, characterized by their secretion of interferon gamma (IFNγ) and interleukin (IL)-17 (IL-17A/F), respectively, have deleterious activities in tissue-specific autoimmune diseases, including inflammatory bowel disease and MS, as demonstrated in mouse models and suggested in human pathology^12,13^. More specifically, so-called pathogenic TH17 cells (also reported as TH1.17 or TH17* in the literature) produce appreciable amounts of GM-CSF, driving clinical symptoms in murine models of MS such as EAE^14,15^. At odds with these pro-inflammatory cells, another subset of CD4^+^ T cells, called regulatory T cells (Treg cells) and characterized by its high expression of the transcription factor Foxp3, is crucial for the suppression of autoimmunity^16^. Hence, depletion of Treg cells exacerbates EAE severity^17^.

Canonical NF-κB subunits are largely implicated in T-cell activation, survival, as well as in the polarization toward the TH17 lineage and the development of autoimmunity^18–20^. Moreover, these proteins are critical for Treg cell development and function^21,22^. In contrast, the T-cell-intrinsic functions of the alternative pathway remain unclear. Mice and patients with germline mutations in *Map3k14* (encoding NIK) or *Relb* exhibit spontaneous T-cell activation and profound immunodeficiency, though this may rely on T-cell-extrinsic mechanisms^23–26^. Tconv cells isolated from mice harboring either germline or T-cell-restricted ablation of *Map3k14*, displayed a normal TH1 and TH2 polarization capacity, but impaired TH17 differentiation in vitro^27,28^. Consequently, *Map3k14* ablation conferred full resistance to EAE. In contrast, experiments using animals with a non-processable form of p100 or with constitutive activation of RelB, suggested that RelB could directly inhibit TH17 differentiation^29,30^. In line with this, *Relb*^–/–^ T cells, though they may have an impaired TH1 differentiation ability in vitro^31^, induced severe EAE upon transfer to immunodeficient recipients, associated with enhanced TH17 cytokines in the inflamed CNS^8^. However, conflicting results were also reported^30^, and the specific contribution of RelB to TH polarization and T-cell mediated autoimmunity is thus still debated. This is largely due to the fact that the study of cell-autonomous functions was so far prevented by the early and lethal autoimmunity arising in mice with germline deletion of *Relb*^26^. To overcome this issue, we used mice carrying specific *Relb* ablation in T cells. We found that T-cell development in vivo, TH polarization in vitro and T-cell-transfer-induced colitis did not require RelB activity. Conversely, the severity of EAE was significantly reduced in mice with *Relb*-deficient T cells, associated with impaired accumulation of GM-CSF^+^ T cells in the inflamed CNS. Our results reveal a discrete contribution of RelB in T-cell biology and highlight this NF-κB subunit as a putative therapeutic target for the treatment of MS.

## Results

### RelB is dispensable for T-cell homeostasis at steady-state

**Figure 1:**
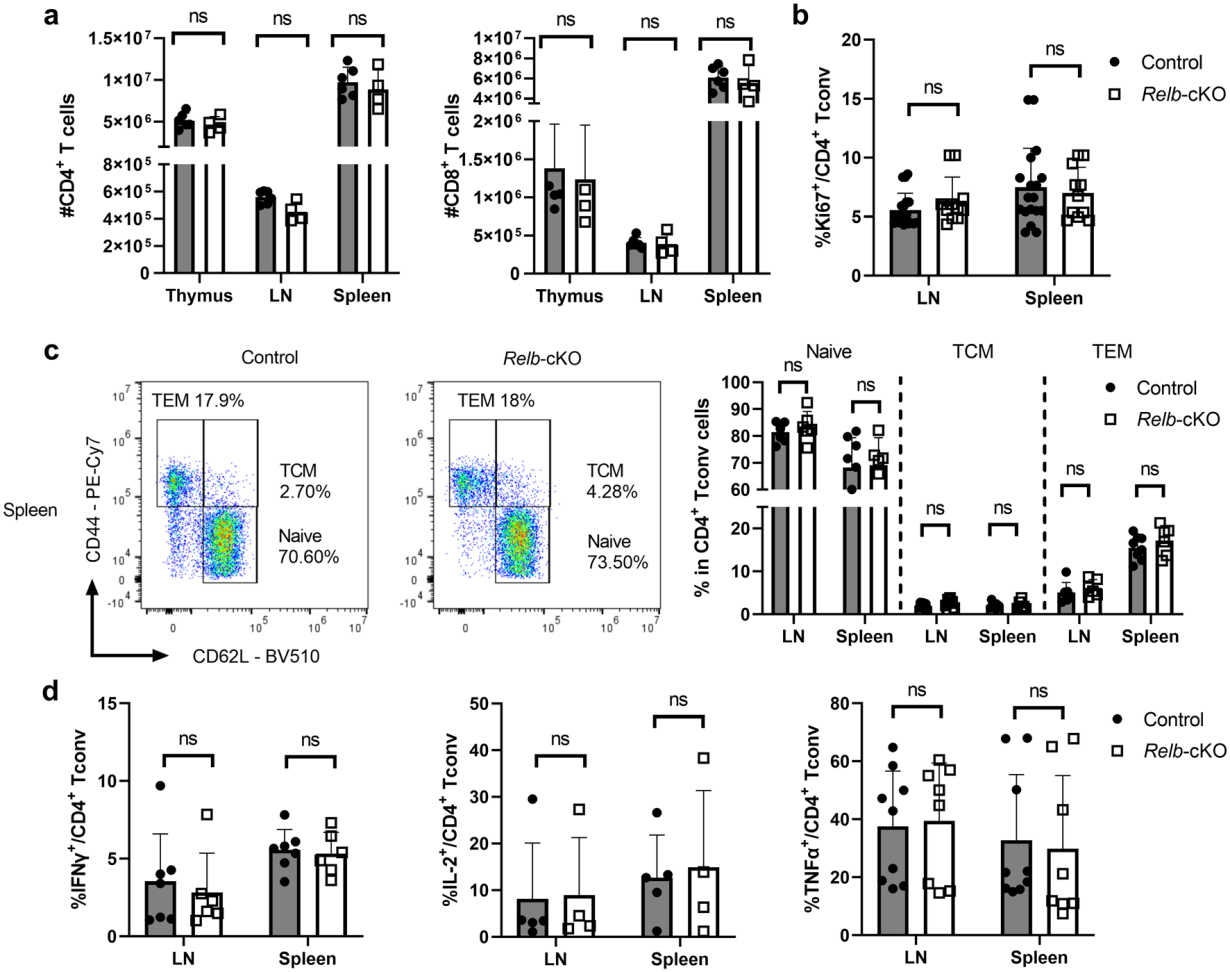
Conditional ablation of *Relb* in T cells does not impair their homeostasis at steady-state. Thymus, spleen and peripheral lymph nodes (LN) from CD4^cre^ (control) and CD4^cre^*Relb*^F/F^ (*Relb*-cKO) mice were analyzed by flow cytometry. (**a**) Quantification of live TCR-β^+^CD4^+^ and CD8^+^ cells. (**b**) Proportion of Ki67^+^ in CD4^+^Foxp3^-^ (Tconv) cells. (**c**) Representative dot plots in the spleen and cumulative data of CD44 and CD62L expression in Tconv cells. T_CM_: T central memory; T_E__M_: T effector memory. (**d**) Cytokine expression by Tconv cells following restimulation with PMA/ionomycin. Each dot represents an individual mouse from 3-5 experiments. Mean + /- SEM is shown. *ns*: non-significant.

To investigate the T-cell-autonomous functions of RelB, we crossed mice carrying *Relb*-floxed alleles with the CD4^cre^ strain, allowing the conditional ablation of *RelB* in T-cells (*Relb*-cKO)^32^. Relb-cKO offspring were viable and mutant mice displayed no signs of autoimmunity up to 12 months after birth (data not shown). Importantly, flow cytometry analyses in adult animals showed that T-cell distribution in the thymus remained unchanged compared to CD4^cre^ control mice (Fig. 1a and data not shown). The proportion and quantity of CD4^+^ and CD8^+^ mature T cells in the spleen and peripheral lymph nodes were not affected by the absence of RelB (Fig. 1a). Furthermore, in vivo activation and proliferation levels, measured through the expression of Ki67, CD44 and CD62L, were normal (Fig. 1b-c). Since NF-κB signaling has been associated with the expression of inflammatory cytokines by T cells, we measured IFN-γ, IL-2 and TNFα expression by Tconv cells following ex vivo polyclonal restimulation of lymphoid tissues. Once again, the proportion of cytokine-producing T cells did not differ between control and *Relb*-cKO mice (Fig. 1d). Together, these data showed that *RelB* ablation did not impact steady-state homeostasis and function of Tconv cells.

### In vitro properties and TH polarization are maintained in the absence of RelB

**Figure 2:**
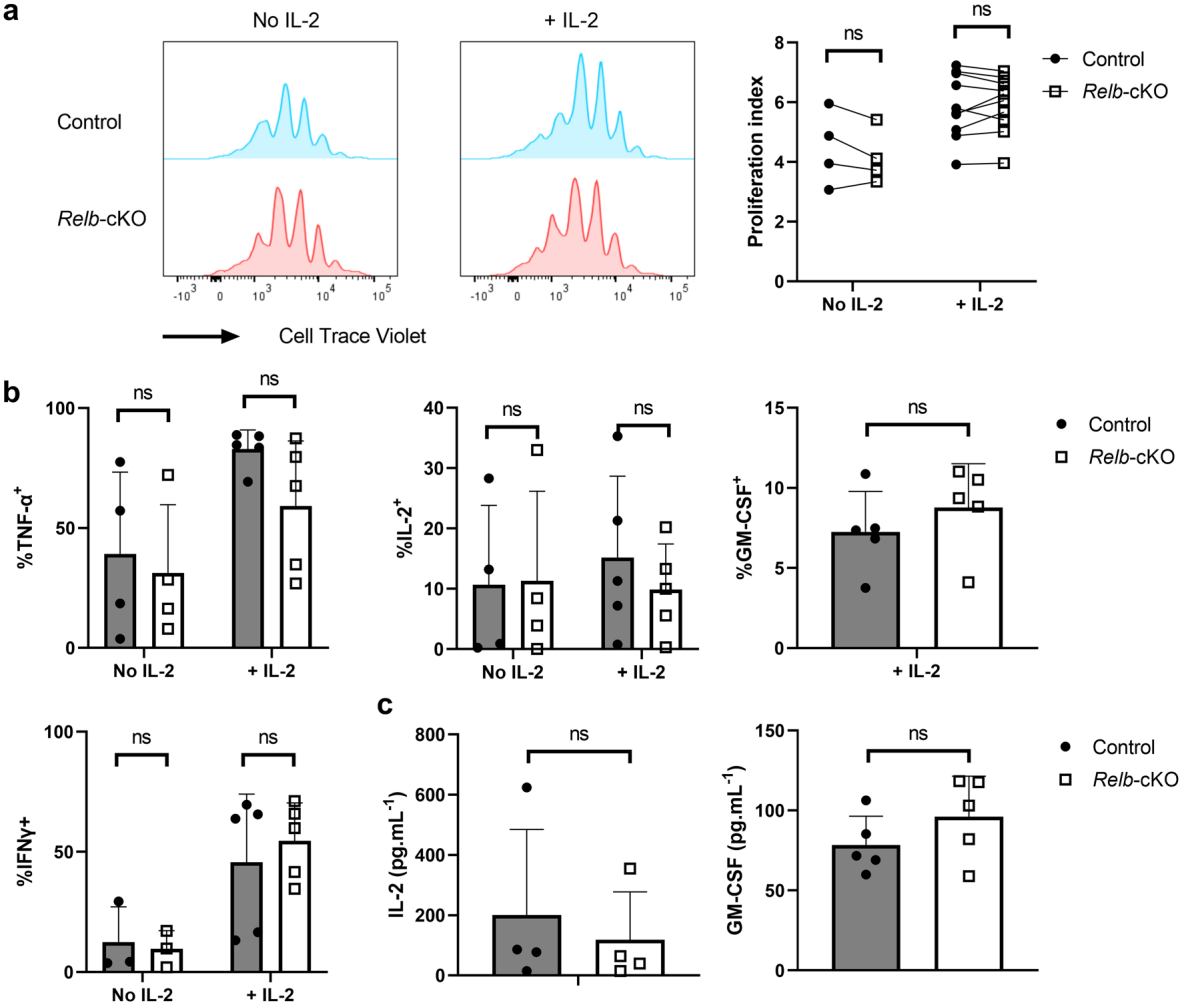
Normal proliferative capacity and cytokine expression of *Relb*-deficient T cells in vitro. CTV-labeled naïve Tconv cells were stimulated with soluble anti-CD3 + mitomycin C-treated feeders. (**a**,**b**) After 4 days of culture, cells were restimulated with PMA/ionomycin and analyzed by flow cytometry. (**a**) Representative CTV profile and cumulative proliferation index of live cells. (**b**) Cytokine expression following restimulation with PMA/ionomycin. (**c**) ELISA analysis of supernatants after 24 h of culture. Each dot represents an individual mouse from 5 experiments. Mean + /- SEM is shown. *ns*: non-significant.

**Figure 3:**
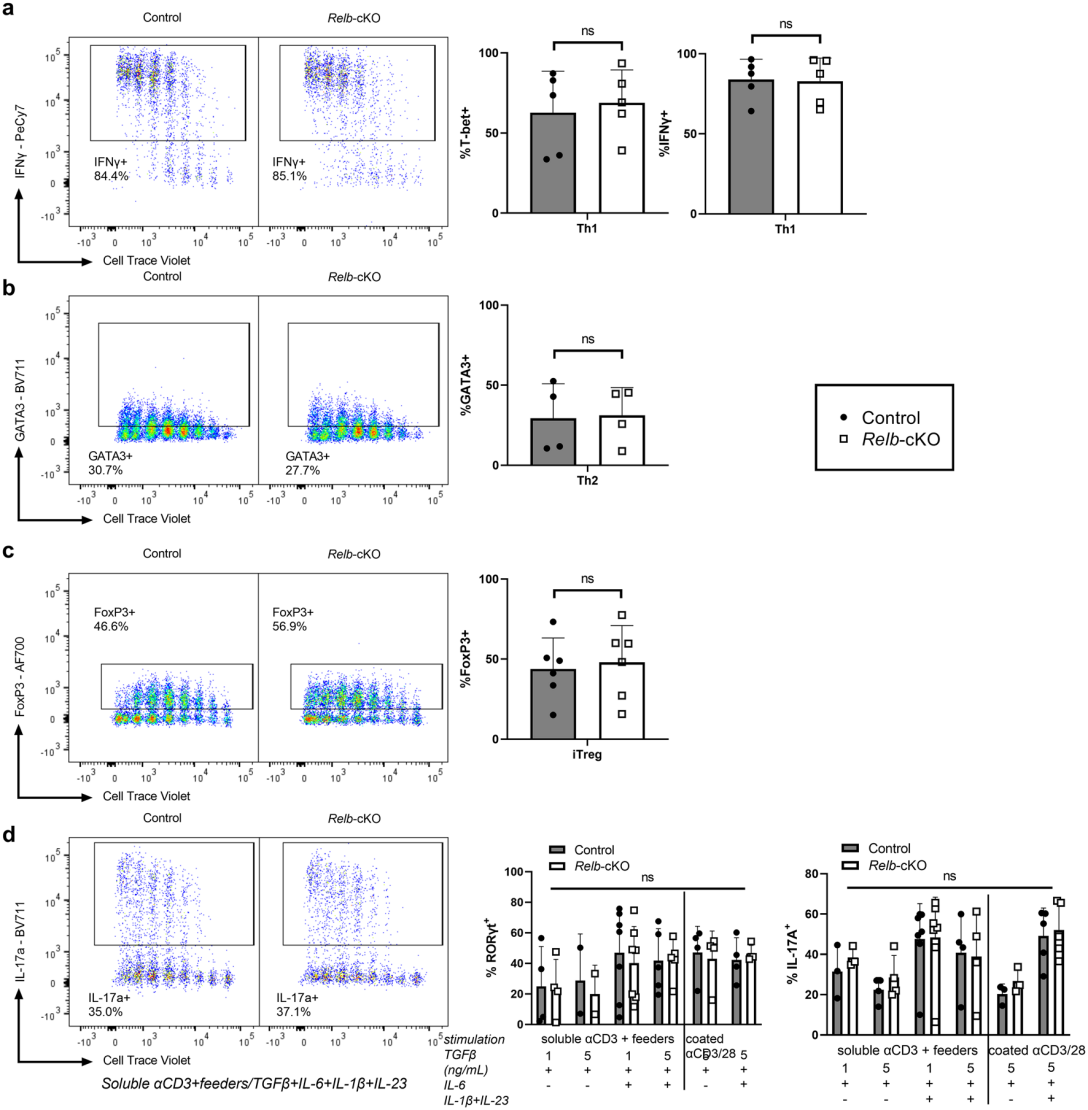
Thelper polarization profile is maintained in the absence of RelB. CTV-labeled naïve Tconv cells were stimulated in TH1, TH2, iTreg-polarizing conditions (**a-c**) or with different TH17 stimulation protocols (**d**) as described in the *Methods* section . After 4 days of culture, cells were restimulated with PMA/ionomycin and analyzed by flow cytometry. (**a**) Percentage of T-bet^+^ and IFNγ^+^ cells in the TH1 condition, (**b**) GATA3^+^ cells in the TH2 condition, and (**c**) Foxp3^+^ cells in the iTreg condition. (**d**) Percentage of RORγt^+^ and IL-17a^+^ cells in various TH17 conditions. *ns*: non-significant.

Next, we evaluated the impact of *Relb* ablation on T-cell behavior in vitro. Naïve Tconv cells isolated from the spleen and LN of control and *Relb*-cKO mice, displayed similar levels of proliferation following CD3/CD28 stimulation, in the presence or absence of IL-2 (Fig. 2a). In the same setting, the proportion of IFNγ^+^, IL-2^+^, TNFα^+^ and GM-CSF^+^ cells was unaltered, as well as the amount of secreted cytokines (Fig. 2b,c). Following stimulation in TH1-, TH2- or iTreg-polarizing conditions, we detected normal expression levels of IFNγ/T-bet, GATA-3, and Foxp3, respectively, suggesting that RelB was expendable for these TH subsets (Fig. 3a–c). Over the last few years, several reports have pondered over the implication of the alternative NF-κB pathway in the biology of TH17 cells. We thus carefully examined the TH17 polarization potential of *Relb*-deficient Tconv cells. Surprisingly, the ability of Tconv cells to convert into IL-17A^+^ and RORγt^+^ TH17 cells when cultured with anti-CD3, feeders, TGF-β and IL-6 was not impacted by the absence of *Relb* (Fig. 3d). Similar results were obtained following the addition of IL-1β and IL-23, and using plate-coated anti-CD3 and -CD28. Thus, though it was described that RelB activation may inhibit IL-17A production by TH17 cells, its ablation did not seem to modify the TH17 polarization capacity of naive Tconv cells.

### RelB expression is not required for T-cell mediated colitis

**Figure 4:**
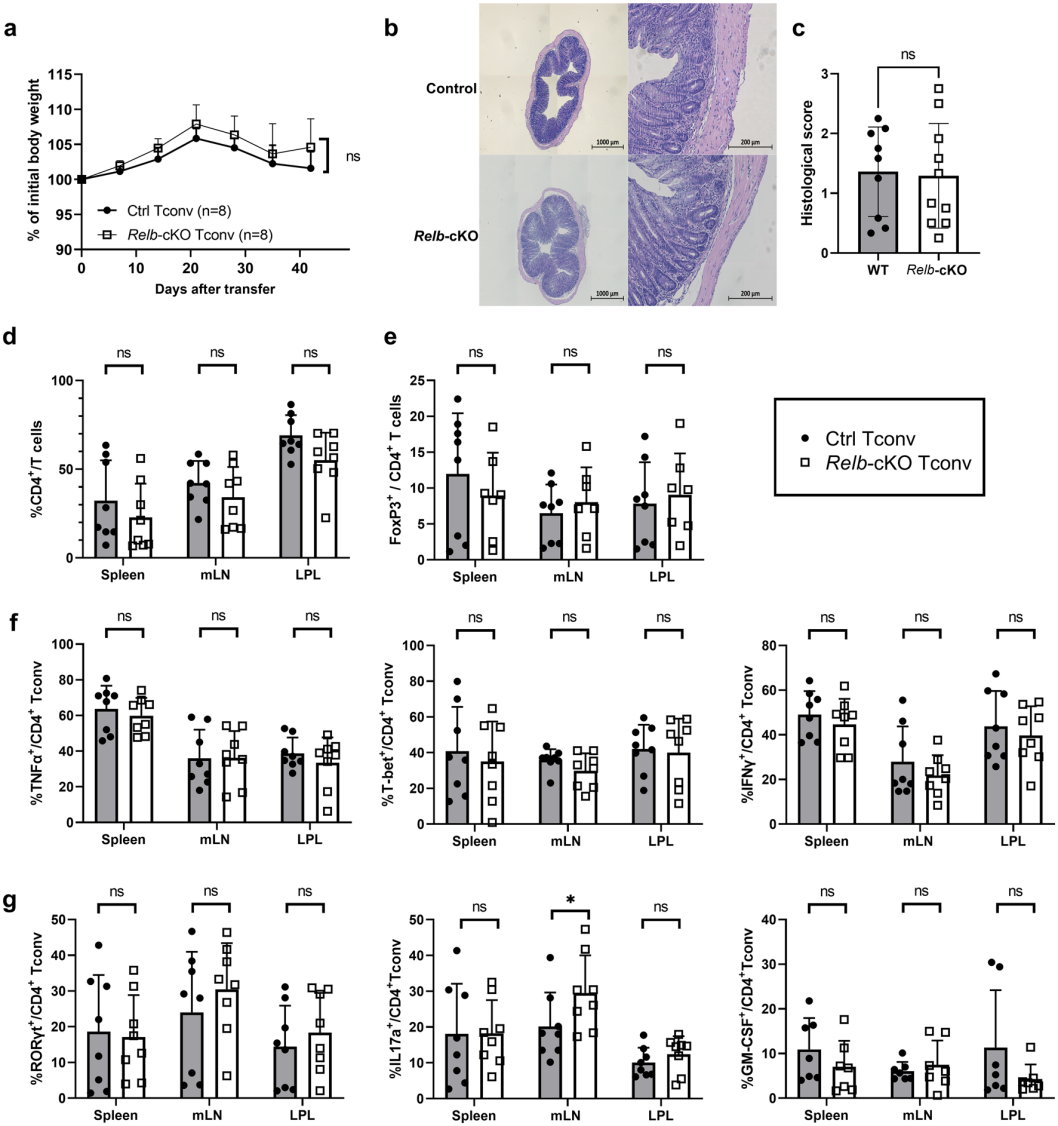
The colitogenic potential of Tconv cells is maintained in the absence of RelB. Naïve Tconv cells were transferred to sex-matched *Rag2*^–/–^ recipients. (**a**) Weight curves presented according to the % of initial body weight. (**b,c**) Histological analysis of colon sections, 6 weeks after transfer. (**b**) Representative section of colon stained with H/E. Bars: original magnification 10X (left), 50X (right). (**c**) Double-blinded histological score. (**d-g**) 6 weeks after transfer, spleens, mesentertic lymph nodes (mLN) and colon lamina propria leukocytes (LPL) were processed for FACS analyses following restimulation with PMA/ionomycin. Each dot represents a mouse from 3 independent experiments; mean + /- SEM is shown. *p < 0.05, *ns*: non-significant.

To test the TH polarization capacity and pathogenic function of *Relb*-deficient T cells in vivo, we next used a T-cell-transfer model of colitis, in which naïve Tconv cells are transferred to immunodeficient *Rag2*^–/–^ recipients. Transfer of control Tconv induced pronounced colon inflammation, leading to moderate weight loss (Fig. 4a). We observed a similar phenotype when *Relb*-deficient Tconv were used, which was also confirmed by histological analyses (Fig. 4b,c). FACS analyses 6 weeks after cell transfer revealed equivalent lymphoid tissue reconstitution and similar colon infiltration by Tconv, suggesting that RelB was dispensable for lymphopenia-induced proliferation and survival in immunocompromised hosts (Fig. 4d). Moreover, the proportion of Foxp3^+^ iTreg cells was unaltered between the two strains, thereby recapitulating our in vitro data (Fig. 4e). The expression of “TH1” markers such as T-Bet, IFNγ or TNFα was also stable (Fig. 4f). Interestingly, whereas the overall proportion of RORγt^+^ T cells was not impacted by the absence of RelB, we detected a significant increase in the percentage of IL-17A^+^ cells specifically in the mesenteric lymph nodes (Fig. 4g). Thus, in this setting, RelB may act as an inhibitor of IL-17A production, as previously reported in other systems. However, the clinical outcome did not differ, presumably because the expression of IL-17A and GM-CSF in the colon was independent of RelB (Fig. 4g). Collectively, this showed that RelB expression was not involved in the pathogenic function of TH cells upon transfer to lymphopenic hosts.

### RelB promotes T-cell pathogenicity in EAE

**Figure 5:**
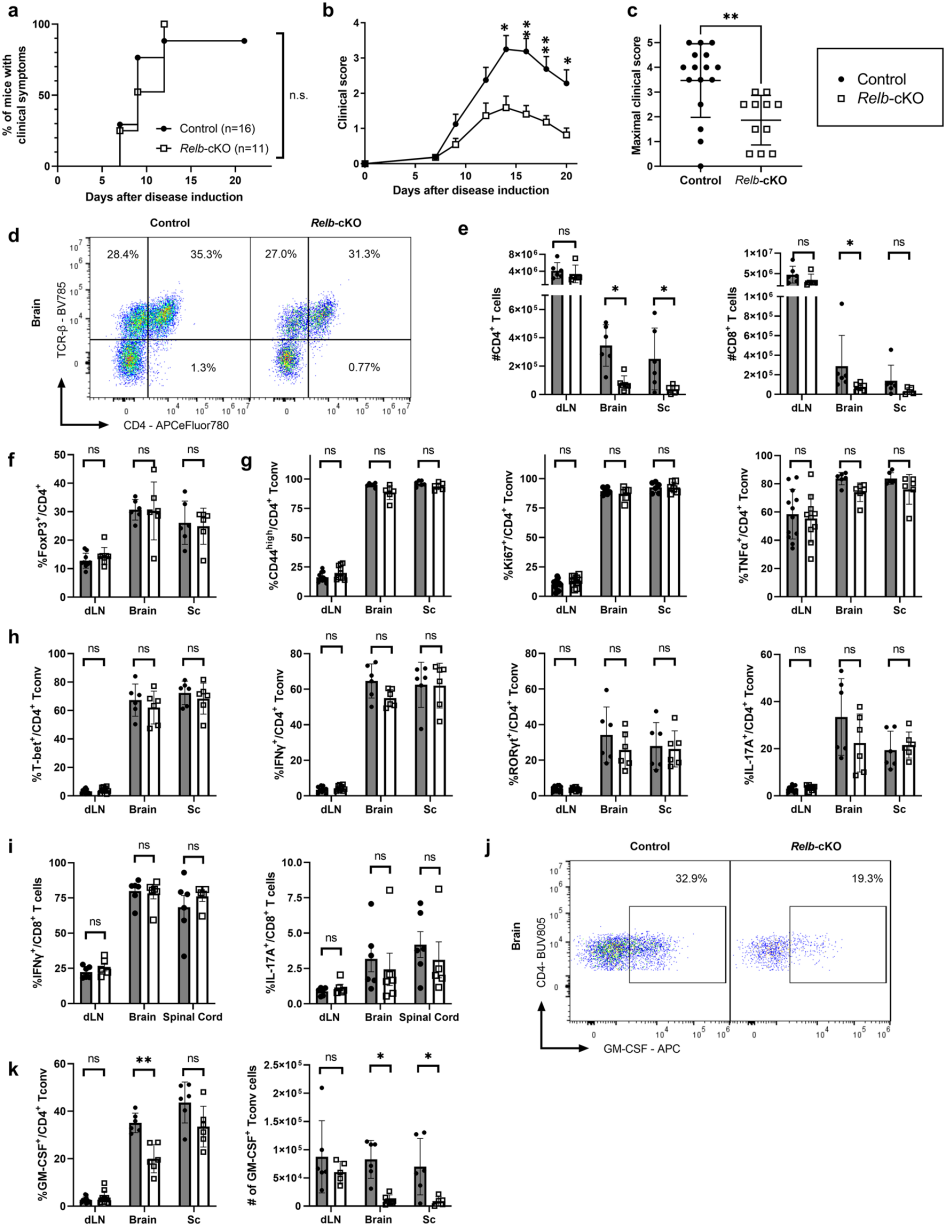
T-cell autonomous RelB is required for optimal pathogenicity in EAE. Active EAE was induced as described in the Methods**.** (**a**) Cumulative incidence of detectable disease (score > 0), (**b**) mean clinical scores and (**c**) maximal scores of each mouse during the course of disease were recorded. (**d-k**) 21 days after transfer, tissues were processed for FACS analyses following restimulation with PMA/ionomycin. (**d**) Representative TCRβ and CD45 staining in gated live brain cells. (**e**) Absolute numbers of CD4^+^ and CD8^+^ T cells (live CD45^+^TCRβ^+^CD4^+^ / CD8^+^) in the indicated tissues. (**f**) Percentage of Treg cells among CD4^+^ T cells. (**g,h**) Percentage of Tconv cells (live CD45^+^TCRβ^+^CD4^+^Foxp3^-^) expressing the indicated markers. (**i**) Percentage of IFNγ and IL-17A expressing CD8^+^ T cells (live CD45^+^TCRβ^+^CD8^+^) (**j**) Representative GM-CSF staining in brain Tconv cells. (**k**) Cumulative proportion and number of live CD45^+^TCRβ^+^CD4^+^Foxp3^-^GM-CSF^+^ Tconv cells. Each dot represents a mouse from 3 independent experiments; mean + /- SEM is shown. *p < 0.05, **p < 0.005, ns: non-significant.

To directly assess the role of RelB in a murine model of T-cell mediated autoimmunity, we immunized control and mutant mice with MOG_33-55_ peptide to induce EAE. As expected, immunization of control mice led to detectable and severe clinical symptoms in most animals, characterized by a peak in the disease 2 weeks after induction, and followed by a spontaneous partial remission (Fig. 5a–c). Interestingly, even though the onset of disease was not delayed in *Relb*-cKO animals, maximal scores were significantly lower than that in the control group, suggesting that RelB may limit the pathogenic function of T cells. To understand this mechanism, we assessed the proportion and phenotype of T cells in the inflamed CNS and draining lymph nodes 20 days after immunization. Although we observed similar numbers of total and CD4^+^ T cells in draining lymph nodes (dLN), accumulation of CD45^+^ cells-and thus, of T cells- was drastically decreased in the brain and spinal cord of *Relb*-cKO animals; however the proportion of T cells among CD45^+^_+_ cells was not modified (Fig. 5d,e). Moreover, the proportion of Foxp3^+^ Treg cells remained intact, suggesting an impairment of the Tconv compartment, rather than an enhanced accumulation of Treg cells (Fig. 5f). The proportion of activated (CD44^hi^) and proliferating (Ki67^+^) cells was equivalent between strains (Fig. 5g), indicative of an intact T-cell priming but an impaired migration toward the CNS, and/or a defective survival in situ. We thus assessed the TH profile of each strain. The expression of TNFα as well as TH1 (T-bet and IFNγ) and classical TH17 (RORγt and IL-17A) markers was unaffected in *Relb*-cKO animals (Fig. 5g,h). IFNγ and IL-17A production by CD8^+^ T cells was not impacted either, suggesting that their function was not altered in the absence of *Relb* (Fig. 5i). We detected a specific and significant decrease in the proportion and numbers of GM-CSF^+^ Tconv cells in the brain of *Relb*-cKO mice (Fig. 5j,k). A similar trend was observed in the inflamed spinal cord, though the values did not reach statistical significance. Overall, the absolute numbers of GM-CSF^+^ Tconv cells, which directly impact the severity of the disease, were dramatically decreased in the brain and spinal cord of mutant mice (Fig. 5k). Altogether, these data highlight a pathogenic T-cell-intrinsic function of RelB in CNS autoimmunity, through its role in GM-CSF expression.

## Discussion

The major roles of the canonical NF-κB pathway in TH17 cell polarization and pathogenic function in autoimmunity are well established. However, despite growing literature, the roles of the alternative pathway and subunits remain poorly defined. This lack of knowledge is partly due to the absence of adequate mouse models to study the cell-autonomous functions of RelB. In the present study, although *Relb* expression was largely dispensable for Tconv biology at steady-state and was not required for TH polarization in vitro, the absence of *Relb* significantly improved clinical symptoms of EAE by limiting the pathogenic function of T cells in the inflamed CNS. These findings further our knowledge on the role of NF-κB transcription factors in immune responses.

Our *Relb*-cKO model was viable, unlike mice carrying a germline deletion of *Relb*, and no autoimmune symptoms or inflammation could be detected in our animals, thus confirming previous literature establishing that the lethal autoimmunity and spontaneous T-cell activation observed in *Relb*^–/–^ mice is independent of the absence of RelB in T cells^33,34^. As such, the phenotype and activation levels of *Relb*-deficient T cells was largely comparable to that of control cells. We next used our *Relb*-cKO strain to fully decipher the cell-autonomous function of RelB in T-cell features in vitro, including cell proliferation, cytokine expression and polarization toward different Thelper lineages. To date, conflicting data have illustrated the putative contribution of alternative NF-κB subunits to TH polarization. For instance, total T cells from *Relb*^–/–^ animals, but not *Nfkb2*^–/–^ or *Map3k14*^–/–^ mice, displayed defects in TH1 polarization^28,30,31^, suggesting an alternative pathway-independent function of RelB. Because we detected intact IFNγ and T-bet expression in TH1 cells from *Relb*-cKO mice, this early observation was likely due to a cell-extrinsic phenomenon. The role of this pathway in in vitro-differentiated TH17 cells is even more debated. Ablation of NIK or p100 reduced IL-17A expression in TH17 cells^28,30^. At odds with these reports, it was shown that forced activation and overexpression of RelB in T cells significantly impaired TH17 polarization. Mechanistically, following OX-40 engagement, RelB could directly bind to the *Il17* locus and recruit histone modifiers to inhibit *Il17a* expression^8^. Conversely, we showed here that T-cell-restricted ablation of *Relb* did not modify the ability of Tconv to polarize into RORγt^+^ IL17-A^+^ cells in vitro. This confirmed the conclusions of a previous report using *Lck*^cre^*Relb*^floxed^ animals^34^. Thus, whereas strong activation of RelB may be deleterious for IL-17A expression, its absence did not impact polarization toward TH17 cells in vitro.

In addition, RelB was dispensable for lymphopenia-induced proliferation and colon inflammation following transfer of Tconv to immunodeficient recipients. This represents an important insight into the pathophysiology of IBD. Indeed, it was shown that NIK activity was integral, both in intestinal epithelial cells and dendritic cells, for the development of IBD in mouse models^35,36^. In addition, *Chuk*^–/–^ T cells (deficient for IKKα), that have reduced activation of the canonical and alternative pathways, fail to induce IBD when transferred to *Rag1*^–/–^ recipients^37^. Our experiments demonstrate that RelB activation per se was not involved in the pathogenic function of T cells in IBD, suggesting a cell type-specific contribution of the alternative NF-κB pathway in intestinal autoimmunity.

Interestingly, T-cell-intrinsic *RelB* expression was critical to induce severe symptoms of EAE. This observation is in stark contrast with previous reports showing either no effect^30^ or a deleterious impact^8^ of *Relb* ablation on the function of T cells during EAE. However, these two studies used cell transfer experiments to address the question. Our results are to our knowledge the first to directly investigate the function of RelB in T cells using an active EAE model in otherwise unmanipulated animals. Thus, similarly to what was observed in oligodendrocytes or dendritic cells, RelB appears to exert a pathogenic effect in T cells during CNS autoimmunity^38,39^.

From our results, RelB seems to be implicated in discrete steps of the pathogenic function of T cells during EAE, rather than being involved in the differentiation of the whole TH17 lineage in vivo. First, we observed a major decrease in the number of CD4^+^ and CD8^+^ T cells, both in the brain and spinal cord of *Relb*-cKO animals, 20 days after EAE induction. Because T-cell numbers and phenotype were intact in the dLN, this suggested an impaired in situ accumulation on T cells in the inflamed tissues. As the proportion of proliferating cells was untouched, this phenomenon was likely due to a defective survival of *Relb*-deficient T cells. This was in accordance with observations made in NIK- deficient animals^28^. In addition to this reduced accumulation, we detected a specific impairment of GM-CSF^+^ Tconv cells in the brain of mutant mice, whereas the proportion of RORγt ^+^ and IL-17A^+^ cells was not significantly altered. Importantly, the Tc1/Tc17 profile of CD8^+^ T cells also remained unchanged. Nevertheless, additional studies are necessary to investigate the potential involvement of RelB in the development and function of the diverse CD8^+^ T-cell subsets that were proposed to exert both immunoregulatory and pathogenic roles in neuroinflammation^40,41^. The precise molecular mechanism underlying this defective GM-CSF expression remains unclear. In contrast to c-Rel and p52, it was proposed that RelB could not directly bind and activate the putative kB-binding site in the *Csf2* promoter in mouse T cells^30^. However, divergent conclusions were reached using human cell lines^42^. It would be of interest to measure the binding of RelB to the *Csf2* locus in CNS-infiltrating Tconv cells during EAE, for instance using ChIP-Seq methods. Nevertheless, the low number of T cells that can be isolated from such non-lymphoid tissues poses a major technical challenge.

It would also be important to elucidate how RelB modulates GM-CSF expression in T cells. RelB was suggested to exert its pleiotropic functions in neuroinflammation through different molecular mechanisms^43^: (i) activation of the alternative NF-κB pathway leading to translocation of p52-RelB dimers, (ii) ‘canonical-like’ association of RelB-p50 and RelB-RelA dimers, and (iii) epigenetic modifications driven by RelB monomers or homodimers. Again, ChIP experiments as well as the use of mouse models with conditional ablation of other NF-κB subunits would be relevant tools to address this specific question.

Collectively, our data offer new insights into the regulation of T-cell immunity by the different members of the NF-κB family. While c-Rel seems critical to kickstart the expression of RORγt and the TH17 lineage^19^, we herein propose that RelB, exerts selective functions in the pathogenic programing of TH cells during ongoing CNS inflammation, rather than in the priming phase of the disease. Thus, we highlight this understudied NF-κB subunit as a putative therapeutic target in EAE and MS.

## Methods

### Mice

*Relb*-Floxed mice were previously described^44^. CD4^cre^ (Tg(CD4-cre)^1Cw1^) on a C57Bl/6 J background were purchased from the Jackson Laboratory. *Rag2*^–/–^ and C57Bl/6 CD45.1 (*Ptprc*^*a*^* Pepc*^*b*^/BoyJ) mice were purchased from Charles River Laboratories France. Mice were bred and used in specific-pathogen-free (SPF) conditions at the CRCL animal facility (Anican). Animals were housed in individually ventilated cages with temperature-controlled conditions under a 12-h-light/dark cycle with free access to drinking water and food. Adult (6–28-week-old) male or female mice were used for all experiments. Studies were conducted in accordance with the animal care guidelines of the European Union and French laws. Protocols were validated by the local Animal Ethic Evaluation Committee (committee C2A15, *Comité d’Evaluation Commun au Centre Léon Bérard, à l’Animalerie de transit de l’ENS, au PBES et au laboratoire P4*) (protocol CLB-2019061914498113). The authors complied with the ARRIVE guidelines.

### Adoptive cell transfer-induced colitis

Donor mice were euthanized by cervical dislocation and spleens and peripheral lymph nodes were harvest in ice cold NaCl. Sterile single cell suspensions were obtained by mechanical dilaceration through a 70 µm cell strainer. Red blood cells were lysed in RBC lysis buffer (NH4Cl 0.154 M + NaHCO3 0.14 M + EDTA 0.1 mM) for 10 min at room temperature. Naïve CD4^+^ T cells were negatively isolated using the Naïve CD4^+^ T Cell Isolation Kit (Miltenyi), with a purity > 90%. Cells were resuspended at 20 × 10^6^ cells/mL in ice cold PBS 1X and 2 × 10^6^ cells were injected intravenously in the retro orbital sinus of recipient *Rag2*^–/–^ mice under isofluorane-induced anesthesia. Mice were weighed once a week.

### EAE

Mice were injected subcutaneously in each flank with 50 μg of MOG_35-55_ peptide (Smart Biosciences SB023) emulsified in 100 μL of complete Freund adjuvant (Sigma-Aldrich) supplemented with 243 μg of heat-killed Mycobacterium tuberculosis H37Ra (Difco-BD Bioscience 2311). Bordetella pertussis toxin (200 ng/injection, Enzo) was injected intravenously at the time of immunization and two days later. Disease severity was evaluated every other day following an established scoring system, in a semi-blinded fashion: 0, no clinical sign; 1, limp tail; 2, limp tail, impaired righting reflex, and paresis of one limb; 3, hindlimb paralysis; 4, complete hindlimb and partial forelimb paralysis; to 5, moribund/death. A score of 5 was permanently attributed to dead animals. After 21 days, mice were anesthetized with a mixture of ketamine and xylazine and perfused with ice-cold PBS1X. Draining (brachial) lymph nodes, brain and spinal cord were harvested in ice cold NaCl solutions.

### Preparation of cell suspensions

Single cell suspensions from LN and spleens were obtained by mechanical dilaceration in FACS Buffer (PBS 1X + 2% FBS, 2 mM EDTA) with glass slides, strained, washed in complete RPMI and enumerated.

Brains and spinal cords were cut into small pieces and digested in RPMI 1640 (Gibco) supplemented with 1 mg/mL collagenase type IV (Sigma-Aldrich), 500 μg/mL DNase I (Sigma-Aldrich) for 30 min at 37 °C followed by mechanical desegregation. Reactions were stopped by the addition of 15 mL PBS1X containing 5 mM EDTA. The solution was passed through a 70 µm cell strainer and residual solid pieces were mechanically disrupted. After centrifugation, cell pellets were resuspended in 8 mL of Percoll 40% (Sigma-Aldrich) and then laid on 4 mL Percoll 80% in a 15 mL polypropylene tube. Tubes were spun at 2500 rpm for 20 min at RT. Mononuclear cells were collected from the interface of the 40:80% Percoll gradient, washed in complete RPMI and enumerated.

Colons were extensively washed and fragments were incubated in 5 mL of cell dissociation solution (Ca^2+^- and Mg^2+^-free containing 5 mM EDTA and 10 mM HEPES) and incubated for 20 min at 37 °C under slow rotation. Supernatant was discarded and fragments were minced and digested in 5 mL PBS containing 5% FBS, 1 mg/mL of Collagenase and 400 µg/mL DNaseI for 20 min at 37 °C under slow rotation. Digestion was stopped by adding 15 mL PBS1X supplemented with EDTA 5 mM. Lamina propria leukocytes (LPL) cells were passed through a 70 µm cell strainer. Mononuclear cells were isolated by a Percoll gradient as above, washed in complete RPMI and enumerated.

### In vitro T-cell proliferation and polarization assays

Naïve CD4^+^ T cells were negatively isolated using the Naïve CD4^+^ T Cell Isolation Kit (Miltenyi). Cells were labeled using the CellTrace Violet Cell Proliferation Kit (CTV; Thermo Fisher Scientific). Cells were cultured in 120 µL of complete IMDM (10% FBS, 25,000 U Penicilin/Streptomycin, 10 mM sodium pyruvate, non-essential amino acids and 50 µM 2-Mercaptoethanol) (Gibco).

To study proliferation and inflammatory cytokine production, 3 × 10^4^ cells were stimulated with 1.5 × 10^4^ T cell-depleted mitomycin C-treated Ly5.1 splenocytes, 0.5 µg/mL soluble anti-CD3ε (clone 145-2C11, BioXcell) with or without 10 ng/mL mIL-2 (Miltenyi). For TH polarization assays, cells were either stimulated as above, or 5 × 10^4^ cells were stimulated with coated anti-CD3ε 1 µg/mL (BioXcell) and anti-CD28 1 µg/mL (clone 37.51, BioXcell). The culture medium was supplemented as follows: TH0: 10 ng/mL mIL-2; TH1: 20 ng/mL mIL-12 (Peprotech), 10 ng/mL mIL-2 (Miltenyi), 2 µg/mL anti-mouse IL-4 (clone 11B11 BD Biosciences); TH2: 30 ng/mL mIL-4 (Peprotech), 2 µg/mL anti-mouse IFNγ (clone XMG1.2 BD Biosciences), 2 µg/mL anti-mouse IL-12 (clone C17.8 BD Biosciences); iTreg: 5 ng/mL hTGF-β (R&D Systems); 10 ng/mL mIL-2; TH17: 1 or 5 ng/mL hTGF-β, 20 ng/mL mIL-6 (Peprotech), 2 µg/mL anti-mouse IFNγ, 2 µg/mL anti-mouse IL-4, with or without 5 ng/mL mIL-1β (Peprotech); 5 ng/mL hIL-23 (Peprotech) and 2 µg/mL anti-mouse IL-2 (clone JES6-1A12, Biolegend).

After 4 days of culture, supernatants were collected and stored at − 80 °C until further use. Proliferation and subset-specific transcription factors and cytokine expression were assessed by FACS.

### Flow cytometry

For cytokine analysis by FACS, single cell suspensions were stimulated for 4 h at 37 °C with 50 ng/mL PMA (Sigma), 1 µg/mL ionomycin (Sigma-Aldrich) and 1X Protein Transport Inhibitor containing Brefeldin A (BD GolgiPlug). Cells were washed in PBS1X and stained with a viability marker for 10 min at RT in the dark. Cells were washed in PBS1X, and incubated with 50 µL surface marker antibody mix for 20 min a 4 °C in the dark. Cells were then washed in PBS1X and fixed and permeabilized using the eBioscience Foxp3/Transcription Factor Staining Buffer Set (Thermo Fisher Scientific) according to manufacturer’s instructions. Cells were washed and incubated with the intracellular marker antibody mix for 20 min at 4 °C in the dark, washed in permabilization buffer and resuspended in FACS Buffer. The complete list of antibodies can be found in Supplementary Table 1. Acquisition was performed on a LSR Fortessa (BD Biosciences) or an Aurora spectral cytometer (Cytek). Data were analyzed with FlowJO software V10.7.2 (Tree Star, www.flowjo.com).

### Histology

Colons were fixed in 4% formaldehyde solution (Sigma) for 1 day and stored at 4 °C in EtOH 70%. They were then embedded in paraffin, sectioned and stained with hematoxylin/eosin (Sigma-Aldrich). Intestinal inflammation was scored in a blinded fashion using a scoring system based on the following criteria: colon length, inflammatory cell infiltrate (severity and extent), crypt hyperplasia, and presence of neutrophils within the crypts, presence of crypt abscesses, erosion, granulation tissues and villous blunting.

### ELISA

ELISA were performed using “ELISA MAX Deluxe Set Mouse IL-2”, and “ELISA MAX Deluxe Set Mouse GM-CSF” (Biolegend) following manufacturer’s instructions.

### Statistics

Statistics were performed using GraphPad Prism Software v9 (https://www.graphpad.com/scientific-software/prism/). Unless mentioned otherwise, two-tailed Mann–Whitney t test was used to calculate statistical significance. P values < 0.05 were considered significant. ns: non-significant; *p < 0.05; **p < 0.01; ***p < 0.001; ****p < 0.0001.

## Supplementary Information

Supplementary Information.

## Data Availability

Flow cytometry and other raw data will be made available upon request.

## References

[1] Hayden MS, Ghosh S (2008). Shared principles in NF-kappaB signaling. Cell.

[2] Voisin A, Grinberg-Bleyer Y (2021). The many-sided contributions of NF-kappaB to T-cell biology in health and disease. Int. Rev. Cell Mol. Biol..

[3] Oeckinghaus A, Hayden MS, Ghosh S (2011). Crosstalk in NF-κB signaling pathways. Nat. Immunol..

[4] Derudder E (2003). RelB/p50 dimers are differentially regulated by tumor necrosis factor-alpha and lymphotoxin-beta receptor activation: critical roles for p100. J. Biol. Chem..

[5] Shih VF (2012). Control of RelB during dendritic cell activation integrates canonical and noncanonical NF-kappaB pathways. Nat. Immunol..

[6] Chen X, El Gazzar M, Yoza BK, McCall CE (2009). The NF-kappaB factor RelB and histone H3 lysine methyltransferase G9a directly interact to generate epigenetic silencing in endotoxin tolerance. J. Biol. Chem..

[7] Yoza BK, Hu JY, Cousart SL, Forrest LM, McCall CE (2006). Induction of RelB participates in endotoxin tolerance. J. Immunol..

[8] Xiao X (2016). The costimulatory receptor OX40 inhibits interleukin-17 expression through activation of repressive chromatin remodeling pathways. Immunity.

[9] Siebenlist U, Brown K, Claudio E (2005). Control of lymphocyte development by nuclear factor-kappaB. Nat Rev Immunol.

[10] Oh H, Ghosh S (2013). NF-κB: roles and Regulation In Different CD4+ T cell subsets. Immunol. Rev..

[11] Ziegler SF (2016). Division of labour by CD4(+) T helper cells. Nat Rev Immunol.

[12] Domingues HS, Mues M, Lassmann H, Wekerle H, Krishnamoorthy G (2010). Functional and pathogenic differences of Th1 and Th17 cells in experimental autoimmune encephalomyelitis. PLoS ONE.

[13] Imam T, Park S, Kaplan MH, Olson MR (2018). Effector T helper cell subsets in inflammatory bowel diseases. Front Immunol.

[14] Komuczki J (2019). Fate-mapping of GM-CSF expression identifies a discrete subset of inflammation-driving T helper cells regulated by cytokines IL-23 and IL-1beta. Immunity.

[15] Lee Y (2012). Induction and molecular signature of pathogenic TH17 cells. Nat. Immunol..

[16] Josefowicz SZ, Lu L-F, Rudensky AY (2012). Regulatory T cells: mechanisms of differentiation and function. Annu. Rev. Immunol..

[17] McGeachy MJ, Stephens LA, Anderton SM (2005). Natural recovery and protection from autoimmune encephalomyelitis: contribution of CD4+CD25+ regulatory cells within the central nervous system. J. Immunol..

[18] Greve, B. *et al.* I kappa B kinase 2/beta deficiency controls expansion of autoreactive T cells and suppresses experimental autoimmune encephalomyelitis. *J. Immunol. (Baltimore, Md.: 1950)* **179**, 179–185, doi:10.4049/jimmunol.179.1.179 (2007). 10.4049/jimmunol.179.1.179 17579036

[19] Ruan Q (2011). The Th17 immune response is controlled by the Rel-RORγ-RORγ T transcriptional axis. J. Exp. Med..

[20] Chen G (2011). The NF-κB transcription factor c-Rel Is required for Th17 effector cell development in experimental autoimmune encephalomyelitis. J. Immunol..

[21] Oh H (2017). An NF-κB transcription factor-dependent, lineage specific transcriptional program promotes regulatory T cell identity and function. Immunity.

[22] Grinberg-Bleyer Y (2017). NF-κB c-Rel Is crucial for the regulatory T cell immune checkpoint in cancer. Cell.

[23] Shinkura R (1999). Alymphoplasia is caused by a point mutation in the mouse gene encoding Nf-kappa b-inducing kinase. Nat. Genet..

[24] Willmann KL (2014). Biallelic loss-of-function mutation in NIK causes a primary immunodeficiency with multifaceted aberrant lymphoid immunity. Nat. Commun..

[25] Sharfe N (2015). The effects of RelB deficiency on lymphocyte development and function. J. Autoimmun..

[26] Weih F (1995). Multiorgan inflammation and hematopoietic abnormalities in mice with a targeted disruption of RelB, a member of the NF-kappa B/Rel family. Cell.

[27] Jin W, Zhou X-F, Yu J, Cheng X, Sun S-C (2009). Regulation of Th17 cell differentiation and EAE induction by MAP3K NIK. Blood.

[28] Li Y (2016). Cell intrinsic role of NF-κB-inducing kinase in regulating T cell-mediated immune and autoimmune responses. Sci. Rep..

[29] Koliesnik IO (2018). RelB regulates Th17 differentiation in a cell-intrinsic manner. Immunobiology.

[30] Yu, J. *et al.* T cell-intrinsic function of the noncanonical NF-κB pathway in the regulation of GM-CSF expression and experimental autoimmune encephalomyelitis pathogenesis. *J. Immunol. (Baltimore, Md.: 1950)* **193**, 422–430. 10.4049/jimmunol.1303237 (2014). 10.4049/jimmunol.1303237 PMC4076693 24899500

[31] Corn, R. A., Hunter, C., Liou, H.-C., Siebenlist, U. & Boothby, M. R. Opposing roles for RelB and Bcl-3 in regulation of T-box expressed in T cells, GATA-3, and Th effector differentiation. *J. Immunol. (Baltimore, Md.: 1950)* **175**, 2102–2110, doi:10.4049/jimmunol.175.4.2102 (2005). 10.4049/jimmunol.175.4.2102 16081776

[32] Grinberg-Bleyer, Y. *et al.* The Alternative NF-κB Pathway in Regulatory T Cell Homeostasis and Suppressive Function. *J. Immunol. (Baltimore, Md.: 1950)* **200**, 2362–2371. 10.4049/jimmunol.1800042 (2018). 10.4049/jimmunol.1800042 PMC5860980 29459403

[33] Li, J. *et al.* Role of the NF-κB Family Member RelB in Regulation of Foxp3+ Regulatory T Cells In Vivo. *J. Immunol. (Baltimore, Md.: 1950)* **200**, 1325–1334. 10.4049/jimmunol.1701310 (2018). 10.4049/jimmunol.1701310 29298831

[34] Powolny-Budnicka I (2011). RelA and RelB transcription factors in distinct thymocyte populations control lymphotoxin-dependent interleukin-17 production in γδ T Cells. Immunity.

[35] Ramakrishnan SK (2019). Intestinal non-canonical NFkappaB signaling shapes the local and systemic immune response. Nat. Commun..

[36] Jie Z (2018). NIK signaling axis regulates dendritic cell function in intestinal immunity and homeostasis. Nat. Immunol..

[37] Chen X (2015). IKKalpha is required for the homeostasis of regulatory T cells and for the expansion of both regulatory and effector CD4 T cells. FASEB J..

[38] Gupta AS (2019). A detrimental role of RelB in mature oligodendrocytes during experimental acute encephalomyelitis. J Neuroinflammation.

[39] Andreas N (2019). RelB deficiency in dendritic cells protects from autoimmune inflammation due to spontaneous accumulation of tissue T regulatory cells. J. Immunol..

[40] Sinha S, Boyden AW, Itani FR, Crawford MP, Karandikar NJ (2015). CD8(+) T-cells as immune regulators of multiple sclerosis. Front. Immunol..

[41] Salou M, Nicol B, Garcia A, Laplaud DA (2015). Involvement of CD8(+) T cells in multiple sclerosis. Front. Immunol..

[42] Sasaki CY, Ghosh P, Longo DL (2011). Recrui(ent of RelB to the Csf2 promoter enhances RelA-mediated transcription of granulocyte-macrophage colony-stimulating factor. J. Biol. Chem..

[43] Mockenhaupt K, Gonsiewski A, Kordula T (2021). RelB and Neuroinflammation. Cells.

[44] De Silva NS (2016). Transcription factors of the alternative NF-κB pathway are required for germinal center B-cell development. Proc. Natl. Acad. Sci. USA.

