# Supplementary material for: A T cell-intrinsic function for NF-κB RelB in experimental autoimmune encephalomyelitis
Source: Sci Rep. 2021 Oct 4;11:19674. doi: 10.1038/s41598-021-99134-x (PMC8490410; doi:10.1038/s41598-021-99134-x)
Supplement: Supplementary file 1 — Supplementary Information. [file 41598_2021_99134_MOESM1_ESM.pdf]

A T cell-intrinsic function for NF-κB RelB in experimental autoimmune encephalomyelitis

Guilhem Lalle[1], Raphaëlle Lautraite[1], Allison Voisin[1], Julie Twardowski[1], Pierre Stéphan[1], Marlène Perrin-Niquet[1], Ramdane Igalouzène[1], Saidi Soudja[1], Julien C. Marie[1], Marc Vocanson[2], Nilushi De Silva[3], Ulf Klein[4], Sankar Ghosh[5] and Yenkel Grinberg-Bleyer[1,*]

[1] Cancer Research Center of Lyon, UMR INSERM 1052, CNRS 5286, Université Claude Bernard Lyon 1, Labex DEVweCAN, Centre Léon Bérard, Lyon, France.

[2] CIRI-Centre International de Recherche en Infectiologie ; INSERM, U1111, Université Claude Bernard Lyon 1, Ecole Normale Supérieure de Lyon, CNRS UMR 5308, Lyon, France.

[3] Immunity and Cancer Department, Institut Curie, Paris-Sciences-et-Lettres Research University, INSERM U932, Paris, France

[4] Division of Haematology & Immunology, Leeds Institute of Medical Research at St. James's, University of Leeds, Leeds, UK.

[5] Department of Microbiology & Immunology, College of Physicians & Surgeons, Columbia University, New York, NY 10032, USA.

[*] Lead contact: Yenkel.grinberg-bleyer@inserm.fr

Reagent or ressource	Source	Identifier
Purified Anti-Mouse CD16 / CD32(2.4G2)	Tonbo Biosciences	70-0161-U500
BUV805 Rat Anti-Mouse CD4 (GK1.5)	BD Biosciences	612900
APC-eFluor 780 anti-mouse CD4 (GK1.5)	Thermo Fisher Scientific	47-0041-82
Alexa Fluor 532 anti-mouse CD8 alpha (53-6.7)	Thermo Fisher Scientific	58-0081-80
Brilliant violet 570 anti-mouse CD8 alpha (53-6.7)	BioLegend	100739
Alexa Fluor 700 anti-mouse CD25 (PC61)	BioLegend	102024
APC-R700 anti-mouse CD25 (PC61)	BD Biosciences	565134
BUV496 Rat Anti-Mouse CD44 (IM7)	BD Biosciences	741057
BUV395 Rat Anti-Mouse CD45 (30-F11)	BD Biosciences	564279
FITC anti mouse CD45 (30-F11)	Thermo Fisher Scientific	11-0451-85
Biotin anti-mouse CD45 (30-F11)	BioLegend	103103
PE anti-mouse CD45.2 (104)	BD Biosciences	560695
Biotin anti-mouse CD45.1 (A20)	BioLegend	110704
Brilliant violet BV510 anti-mouse CD62L (MEL-14)	BioLegend	B259984
Alexa Fluor 700 anti-mouse CD103 (2E7)	BioLegend	121441
BUV661 anti-mouseCD183 (CXCR3) (CXCR3-173)	BD Biosciences	741681
APC anti-mouse CD196 (CCR6) (22-9L17)	BioLegend	129814
Brilliant Violet 785 anti-mouse TCR β (H57-597)	BioLegend	109249
PerCP/Cyanine5.5 anti-mouse TCR β (H57-597)	BD Biosciences	560657
APC anti-mouse FoxP3 (FJK-16s)	Thermo Fisher Scientific	17-5773-82
eFluor 450 anti-mouse FOXP3 (FJK-16s)	Thermo Fisher Scientific	48-5773-82
PE anti-mouse/human ROR gamma (t) (AFKJS-9)	Thermo Fisher Scientific	12-6988-82
Pe-Cyanine 7 anti mouse T-bet (4B10)	Thermo Fisher Scientific	25-5825-82
PE anti-mouse/human T-bet (4B10)	BioLegend	644809
Brilliant violet 711 anti-GATA3 (L50-823)	BD Biosciences	565449
PerCP/Cyanine5.5 anti-mouse Ki67 (16A8)	BioLegend	652423
Brilliant violet 711 anti-mouse IL-17a (TC11-18H10.1)	BioLegend	506941
Brilliant violet 421 anti-mouse IFNg (XMG1.2)	BioLegend	505830
Alexa Fluor 700 anti-mouse IFNg (XMG1.2)	BD Biosciences	557998
PE-Cyanine 7 anti-mouse IFNg (XMG1.2)	Thermo Fisher Scientific	25-7311-82
APC/Fire 750 anti-mouse GM-CSF (MP1-22E9)	BioLegend	505424
APC anti-mouse GM-CSF (MP1-22E9)	Thermo Fisher Scientific	17-7331-82
Brillant violet 510 anti-mouse IL-17a (TC11-18H10)	BioLegend	564168
Biotin anti-mouse TNF alpha (MP6-XT22)	BioLegend	506311
eFluor 450 anti-mouse TNF alpha (MP6-XT22)	Thermo Fisher Scientific	48-7321-82
PE anti-mouse IL-2 (JES6-5H4)	Thermo Fisher Scientific	12-7021-82
Brilliant violet 711 anti mouse IL-2 (JES6-5H4)	BioLegend	503837
Zombie UV Fixable Viability Kit	BioLegend	423107
Zombie Green Fixable Viability Kit	BioLegend	423111
LIVE/DEAD Fixable Aqua Dead Cell Stain Kit	Thermo Fisher Scientific	L34957
Brilliant Violet 650 Streptavidin	BioLegend	405231
PE Streptavidin	BioLegend	B291758
CellTrace Violet Cell Proliferation Kit	Thermo Fisher Scientific	C34557

Supplementary Table 1. FACS Abs and labels used in this study
